# Supplementary material for: Modeling glioblastoma heterogeneity as a dynamic network of cell states
Source: Mol Syst Biol. 2021 Sep 16;17(9):e10105. doi: 10.15252/msb.202010105 (PMC8444284; doi:10.15252/msb.202010105)
Supplement: Supplementary file 5 — Source Data for Figure 3 [file MSB-17-e10105-s001.zip › Figure3A_sourcedata/GSEA_3065/nosigdb_state5.GseaPreranked.1622718435914/NB_KI_CLUSTER8.html]

Details for gene set NB\_KI\_CLUSTER8[GSEA]

|  || Dataset | state5 |
| Phenotype | NoPhenotypeAvailable |
| Upregulated in class | na\_pos |
| GeneSet | NB\_KI\_CLUSTER8 |
| Enrichment Score (ES) | 0.45430884 |
| Normalized Enrichment Score (NES) | 2.0752795 |
| Nominal p-value | 0.0 |
| FDR q-value | 7.9761905E-4 |
| FWER p-Value | 0.003 |
Table: GSEA Results Summary

  

Fig 1: Enrichment plot: NB\_KI\_CLUSTER8      
 Profile of the Running ES Score & Positions of GeneSet Members on the Rank Ordered List

  

| PROBE | GENE SYMBOL | GENE\_TITLE | RANK IN GENE LIST | RANK METRIC SCORE | RUNNING ES | CORE ENRICHMENT || 1 | WSB1 |  |  | 13 | 0.861 | 0.0146 | Yes |
| 2 | MDM4 |  |  | 21 | 0.811 | 0.0290 | Yes |
| 3 | NKTR |  |  | 42 | 0.700 | 0.0398 | Yes |
| 4 | TMEM259 |  |  | 59 | 0.613 | 0.0494 | Yes |
| 5 | DDX17 |  |  | 64 | 0.595 | 0.0600 | Yes |
| 6 | ZNF75A |  |  | 73 | 0.572 | 0.0698 | Yes |
| 7 | ZNF431 |  |  | 75 | 0.566 | 0.0803 | Yes |
| 8 | PCSK7 |  |  | 86 | 0.545 | 0.0893 | Yes |
| 9 | LENG8 |  |  | 89 | 0.543 | 0.0993 | Yes |
| 10 | FTX |  |  | 99 | 0.529 | 0.1081 | Yes |
| 11 | TTC14 |  |  | 100 | 0.526 | 0.1180 | Yes |
| 12 | CD46 |  |  | 104 | 0.523 | 0.1274 | Yes |
| 13 | PGAP1 |  |  | 125 | 0.503 | 0.1345 | Yes |
| 14 | ARID1B |  |  | 133 | 0.497 | 0.1430 | Yes |
| 15 | CCDC14 |  |  | 136 | 0.492 | 0.1520 | Yes |
| 16 | N4BP2L2 |  |  | 142 | 0.489 | 0.1606 | Yes |
| 17 | RBM6 |  |  | 168 | 0.468 | 0.1665 | Yes |
| 18 | MFSD14C |  |  | 182 | 0.454 | 0.1735 | Yes |
| 19 | STIM2 |  |  | 210 | 0.433 | 0.1784 | Yes |
| 20 | SLC3A2 |  |  | 222 | 0.424 | 0.1851 | Yes |
| 21 | RDH11 |  |  | 224 | 0.422 | 0.1929 | Yes |
| 22 | POGZ |  |  | 234 | 0.417 | 0.1997 | Yes |
| 23 | PABPN1 |  |  | 237 | 0.414 | 0.2072 | Yes |
| 24 | PILRB |  |  | 274 | 0.397 | 0.2104 | Yes |
| 25 | SUN1 |  |  | 292 | 0.387 | 0.2157 | Yes |
| 26 | RBM5 |  |  | 301 | 0.382 | 0.2220 | Yes |
| 27 | CPD |  |  | 317 | 0.374 | 0.2272 | Yes |
| 28 | TRIO |  |  | 323 | 0.372 | 0.2336 | Yes |
| 29 | TTC17 |  |  | 338 | 0.364 | 0.2388 | Yes |
| 30 | APMAP |  |  | 350 | 0.361 | 0.2443 | Yes |
| 31 | UBE2G2 |  |  | 410 | 0.337 | 0.2438 | Yes |
| 32 | TRA2A |  |  | 419 | 0.335 | 0.2491 | Yes |
| 33 | EPM2AIP1 |  |  | 420 | 0.335 | 0.2554 | Yes |
| 34 | HCG18 |  |  | 449 | 0.326 | 0.2583 | Yes |
| 35 | ZNF791 |  |  | 488 | 0.315 | 0.2598 | Yes |
| 36 | GPATCH2L |  |  | 501 | 0.311 | 0.2642 | Yes |
| 37 | OFD1 |  |  | 502 | 0.311 | 0.2700 | Yes |
| 38 | BRAF |  |  | 521 | 0.306 | 0.2737 | Yes |
| 39 | MED17 |  |  | 535 | 0.301 | 0.2778 | Yes |
| 40 | CDK13 |  |  | 541 | 0.300 | 0.2829 | Yes |
| 41 | TMEM245 |  |  | 561 | 0.295 | 0.2862 | Yes |
| 42 | GTF2H2 |  |  | 579 | 0.290 | 0.2897 | Yes |
| 43 | DNAJB14 |  |  | 582 | 0.290 | 0.2949 | Yes |
| 44 | ABCA5 |  |  | 616 | 0.282 | 0.2963 | Yes |
| 45 | GIGYF1 |  |  | 621 | 0.281 | 0.3011 | Yes |
| 46 | CDK10 |  |  | 625 | 0.280 | 0.3060 | Yes |
| 47 | ZMYM2 |  |  | 673 | 0.270 | 0.3056 | Yes |
| 48 | VPS13A |  |  | 680 | 0.268 | 0.3100 | Yes |
| 49 | PRPF38B |  |  | 714 | 0.259 | 0.3110 | Yes |
| 50 | HNRNPH1 |  |  | 736 | 0.253 | 0.3133 | Yes |
| 51 | ZNF638 |  |  | 753 | 0.250 | 0.3162 | Yes |
| 52 | KIAA1109 |  |  | 759 | 0.249 | 0.3202 | Yes |
| 53 | VPS13C |  |  | 761 | 0.249 | 0.3248 | Yes |
| 54 | FRYL |  |  | 762 | 0.248 | 0.3294 | Yes |
| 55 | GPR155 |  |  | 785 | 0.244 | 0.3315 | Yes |
| 56 | FAM131A |  |  | 787 | 0.243 | 0.3359 | Yes |
| 57 | PHTF2 |  |  | 790 | 0.243 | 0.3402 | Yes |
| 58 | MAT2A |  |  | 794 | 0.242 | 0.3444 | Yes |
| 59 | MACF1 |  |  | 805 | 0.240 | 0.3477 | Yes |
| 60 | SHPRH |  |  | 819 | 0.238 | 0.3507 | Yes |
| 61 | ZNF430 |  |  | 835 | 0.235 | 0.3533 | Yes |
| 62 | SLC35A3 |  |  | 841 | 0.234 | 0.3571 | Yes |
| 63 | AAK1 |  |  | 853 | 0.232 | 0.3602 | Yes |
| 64 | ANKRD36 |  |  | 854 | 0.232 | 0.3646 | Yes |
| 65 | PTPN4 |  |  | 861 | 0.230 | 0.3682 | Yes |
| 66 | ANKZF1 |  |  | 879 | 0.228 | 0.3705 | Yes |
| 67 | OGA |  |  | 907 | 0.224 | 0.3716 | Yes |
| 68 | NEMF |  |  | 909 | 0.224 | 0.3756 | Yes |
| 69 | WEE1 |  |  | 919 | 0.222 | 0.3788 | Yes |
| 70 | PRKD3 |  |  | 927 | 0.220 | 0.3821 | Yes |
| 71 | FAM53C |  |  | 932 | 0.220 | 0.3857 | Yes |
| 72 | SNRNP70 |  |  | 935 | 0.220 | 0.3896 | Yes |
| 73 | CLK4 |  |  | 951 | 0.218 | 0.3920 | Yes |
| 74 | ATP2B1 |  |  | 953 | 0.218 | 0.3959 | Yes |
| 75 | INSR |  |  | 993 | 0.212 | 0.3954 | Yes |
| 76 | ODF2L |  |  | 1003 | 0.210 | 0.3983 | Yes |
| 77 | PCNX4 |  |  | 1027 | 0.208 | 0.3995 | Yes |
| 78 | MKLN1 |  |  | 1028 | 0.207 | 0.4034 | Yes |
| 79 | DDHD1 |  |  | 1033 | 0.206 | 0.4068 | Yes |
| 80 | ATXN1 |  |  | 1062 | 0.203 | 0.4074 | Yes |
| 81 | RICTOR |  |  | 1064 | 0.203 | 0.4111 | Yes |
| 82 | TNRC6B |  |  | 1080 | 0.201 | 0.4131 | Yes |
| 83 | PIGQ |  |  | 1085 | 0.200 | 0.4164 | Yes |
| 84 | REV3L |  |  | 1140 | 0.193 | 0.4138 | Yes |
| 85 | CCDC66 |  |  | 1176 | 0.188 | 0.4133 | Yes |
| 86 | XRRA1 |  |  | 1187 | 0.187 | 0.4156 | Yes |
| 87 | RALGAPA1 |  |  | 1201 | 0.185 | 0.4176 | Yes |
| 88 | PHC3 |  |  | 1202 | 0.185 | 0.4210 | Yes |
| 89 | SORL1 |  |  | 1247 | 0.180 | 0.4193 | Yes |
| 90 | MPHOSPH9 |  |  | 1256 | 0.178 | 0.4217 | Yes |
| 91 | CALCOCO1 |  |  | 1316 | 0.169 | 0.4181 | Yes |
| 92 | HTT |  |  | 1317 | 0.169 | 0.4213 | Yes |
| 93 | ELOVL5 |  |  | 1320 | 0.169 | 0.4242 | Yes |
| 94 | NCSTN |  |  | 1348 | 0.166 | 0.4242 | Yes |
| 95 | AP3M2 |  |  | 1352 | 0.166 | 0.4269 | Yes |
| 96 | PHACTR4 |  |  | 1355 | 0.165 | 0.4298 | Yes |
| 97 | NUMA1 |  |  | 1360 | 0.165 | 0.4324 | Yes |
| 98 | SF3B1 |  |  | 1362 | 0.165 | 0.4354 | Yes |
| 99 | CLASRP |  |  | 1367 | 0.164 | 0.4380 | Yes |
| 100 | CDK12 |  |  | 1370 | 0.164 | 0.4409 | Yes |
| 101 | GLMN |  |  | 1375 | 0.164 | 0.4435 | Yes |
| 102 | TRMT13 |  |  | 1406 | 0.161 | 0.4430 | Yes |
| 103 | PRDM2 |  |  | 1407 | 0.161 | 0.4460 | Yes |
| 104 | CDS2 |  |  | 1408 | 0.161 | 0.4490 | Yes |
| 105 | ANKRD26 |  |  | 1454 | 0.157 | 0.4468 | Yes |
| 106 | UBR5 |  |  | 1500 | 0.152 | 0.4444 | Yes |
| 107 | DIP2A |  |  | 1511 | 0.151 | 0.4461 | Yes |
| 108 | CYHR1 |  |  | 1519 | 0.150 | 0.4481 | Yes |
| 109 | SRSF6 |  |  | 1558 | 0.146 | 0.4465 | Yes |
| 110 | RNF168 |  |  | 1605 | 0.141 | 0.4438 | Yes |
| 111 | DPF2 |  |  | 1648 | 0.138 | 0.4416 | Yes |
| 112 | RNF145 |  |  | 1716 | 0.132 | 0.4363 | Yes |
| 113 | STX16 |  |  | 1718 | 0.132 | 0.4387 | Yes |
| 114 | MLLT6 |  |  | 1733 | 0.131 | 0.4395 | Yes |
| 115 | RABL2B |  |  | 1753 | 0.129 | 0.4398 | Yes |
| 116 | ZC3H7A |  |  | 1774 | 0.128 | 0.4398 | Yes |
| 117 | LTN1 |  |  | 1788 | 0.127 | 0.4407 | Yes |
| 118 | BTBD7 |  |  | 1792 | 0.127 | 0.4428 | Yes |
| 119 | QTRT1 |  |  | 1795 | 0.127 | 0.4449 | Yes |
| 120 | CDC42BPA |  |  | 1817 | 0.125 | 0.4448 | Yes |
| 121 | UBR2 |  |  | 1848 | 0.123 | 0.4437 | Yes |
| 122 | ZNF7 |  |  | 1869 | 0.121 | 0.4436 | Yes |
| 123 | KMT2A |  |  | 1876 | 0.120 | 0.4452 | Yes |
| 124 | CEP85L |  |  | 1878 | 0.120 | 0.4473 | Yes |
| 125 | LMBRD1 |  |  | 1879 | 0.120 | 0.4496 | Yes |
| 126 | RNF19A |  |  | 1887 | 0.120 | 0.4510 | Yes |
| 127 | CREBRF |  |  | 1891 | 0.119 | 0.4529 | Yes |
| 128 | HMGCS1 |  |  | 1899 | 0.118 | 0.4543 | Yes |
| 129 | GCC2 |  |  | 1949 | 0.115 | 0.4508 | No |
| 130 | SCRN2 |  |  | 1954 | 0.115 | 0.4525 | No |
| 131 | BIRC6 |  |  | 1988 | 0.112 | 0.4508 | No |
| 132 | PRMT2 |  |  | 2020 | 0.110 | 0.4493 | No |
| 133 | PPM1K |  |  | 2058 | 0.108 | 0.4470 | No |
| 134 | IFRD1 |  |  | 2073 | 0.107 | 0.4474 | No |
| 135 | CDK11A |  |  | 2074 | 0.107 | 0.4494 | No |
| 136 | TRIM8 |  |  | 2078 | 0.107 | 0.4511 | No |
| 137 | YTHDC2 |  |  | 2104 | 0.105 | 0.4502 | No |
| 138 | MPDZ |  |  | 2119 | 0.104 | 0.4505 | No |
| 139 | BTAF1 |  |  | 2143 | 0.102 | 0.4498 | No |
| 140 | DGKZ |  |  | 2147 | 0.102 | 0.4513 | No |
| 141 | MECP2 |  |  | 2162 | 0.101 | 0.4516 | No |
| 142 | HNRNPU |  |  | 2204 | 0.098 | 0.4487 | No |
| 143 | TOM1L2 |  |  | 2214 | 0.097 | 0.4495 | No |
| 144 | PPP1R10 |  |  | 2270 | 0.094 | 0.4449 | No |
| 145 | TRA2B |  |  | 2285 | 0.093 | 0.4451 | No |
| 146 | RANBP2 |  |  | 2287 | 0.093 | 0.4467 | No |
| 147 | CDK11B |  |  | 2300 | 0.092 | 0.4470 | No |
| 148 | RABGAP1 |  |  | 2314 | 0.091 | 0.4472 | No |
| 149 | CPNE1 |  |  | 2338 | 0.090 | 0.4462 | No |
| 150 | MEF2A |  |  | 2349 | 0.089 | 0.4467 | No |
| 151 | KLF12 |  |  | 2355 | 0.088 | 0.4478 | No |
| 152 | CENPC |  |  | 2392 | 0.086 | 0.4453 | No |
| 153 | IVNS1ABP |  |  | 2393 | 0.086 | 0.4469 | No |
| 154 | CHD2 |  |  | 2428 | 0.084 | 0.4445 | No |
| 155 | ATF7IP |  |  | 2439 | 0.083 | 0.4449 | No |
| 156 | GAN |  |  | 2522 | 0.078 | 0.4369 | No |
| 157 | SPTAN1 |  |  | 2534 | 0.077 | 0.4371 | No |
| 158 | PRPF3 |  |  | 2562 | 0.076 | 0.4354 | No |
| 159 | HERC1 |  |  | 2576 | 0.075 | 0.4353 | No |
| 160 | KIAA0355 |  |  | 2704 | 0.068 | 0.4220 | No |
| 161 | FBXO42 |  |  | 2740 | 0.066 | 0.4192 | No |
| 162 | CYTH1 |  |  | 2805 | 0.062 | 0.4130 | No |
| 163 | MSL1 |  |  | 2811 | 0.062 | 0.4135 | No |
| 164 | CWF19L2 |  |  | 2843 | 0.061 | 0.4111 | No |
| 165 | SEC22C |  |  | 2854 | 0.060 | 0.4111 | No |
| 166 | SACM1L |  |  | 2856 | 0.060 | 0.4121 | No |
| 167 | SYNE2 |  |  | 2881 | 0.058 | 0.4104 | No |
| 168 | SRRT |  |  | 2930 | 0.056 | 0.4059 | No |
| 169 | FAR1 |  |  | 3000 | 0.052 | 0.3989 | No |
| 170 | MYO9A |  |  | 3039 | 0.050 | 0.3955 | No |
| 171 | PRPF4B |  |  | 3071 | 0.048 | 0.3928 | No |
| 172 | CAPN7 |  |  | 3085 | 0.048 | 0.3922 | No |
| 173 | XRN1 |  |  | 3095 | 0.047 | 0.3921 | No |
| 174 | ICE2 |  |  | 3105 | 0.047 | 0.3919 | No |
| 175 | SENP5 |  |  | 3115 | 0.046 | 0.3917 | No |
| 176 | INO80D |  |  | 3168 | 0.043 | 0.3866 | No |
| 177 | ZNF675 |  |  | 3213 | 0.041 | 0.3822 | No |
| 178 | MRFAP1L1 |  |  | 3222 | 0.040 | 0.3821 | No |
| 179 | RBBP6 |  |  | 3253 | 0.038 | 0.3793 | No |
| 180 | PUM2 |  |  | 3256 | 0.038 | 0.3798 | No |
| 181 | CCNG2 |  |  | 3262 | 0.038 | 0.3800 | No |
| 182 | PIAS1 |  |  | 3269 | 0.038 | 0.3800 | No |
| 183 | TFDP2 |  |  | 3271 | 0.038 | 0.3806 | No |
| 184 | PRKX |  |  | 3278 | 0.038 | 0.3806 | No |
| 185 | LATS1 |  |  | 3348 | 0.034 | 0.3733 | No |
| 186 | CHORDC1 |  |  | 3360 | 0.033 | 0.3726 | No |
| 187 | PURB |  |  | 3408 | 0.031 | 0.3678 | No |
| 188 | PPP4R3A |  |  | 3485 | 0.028 | 0.3595 | No |
| 189 | EVL |  |  | 3508 | 0.027 | 0.3575 | No |
| 190 | MLLT3 |  |  | 3637 | 0.022 | 0.3432 | No |
| 191 | UBR1 |  |  | 3657 | 0.022 | 0.3414 | No |
| 192 | USE1 |  |  | 3687 | 0.021 | 0.3384 | No |
| 193 | PIK3C2A |  |  | 3769 | 0.017 | 0.3294 | No |
| 194 | ZBTB44 |  |  | 3845 | 0.014 | 0.3210 | No |
| 195 | SEC22B |  |  | 3938 | 0.010 | 0.3106 | No |
| 196 | SNRK |  |  | 3952 | 0.009 | 0.3093 | No |
| 197 | RBM14 |  |  | 3995 | 0.007 | 0.3046 | No |
| 198 | TLE3 |  |  | 4004 | 0.007 | 0.3038 | No |
| 199 | DXO |  |  | 4026 | 0.006 | 0.3015 | No |
| 200 | IDS |  |  | 4033 | 0.006 | 0.3009 | No |
| 201 | EXOSC10 |  |  | 4038 | 0.006 | 0.3006 | No |
| 202 | PDE9A |  |  | 4040 | 0.006 | 0.3006 | No |
| 203 | KIAA2026 |  |  | 4068 | 0.004 | 0.2975 | No |
| 204 | ELMSAN1 |  |  | 4074 | 0.004 | 0.2970 | No |
| 205 | MKNK2 |  |  | 4083 | 0.004 | 0.2962 | No |
| 206 | EPS15 |  |  | 4097 | 0.003 | 0.2947 | No |
| 207 | CCAR2 |  |  | 4121 | 0.003 | 0.2921 | No |
| 208 | PHF1 |  |  | 4206 | -0.002 | 0.2825 | No |
| 209 | SETD2 |  |  | 4219 | -0.002 | 0.2811 | No |
| 210 | CDKN2AIP |  |  | 4245 | -0.003 | 0.2783 | No |
| 211 | CLASP1 |  |  | 4261 | -0.004 | 0.2767 | No |
| 212 | GNB5 |  |  | 4289 | -0.005 | 0.2737 | No |
| 213 | JADE1 |  |  | 4324 | -0.007 | 0.2699 | No |
| 214 | TFCP2 |  |  | 4336 | -0.007 | 0.2687 | No |
| 215 | PAK1 |  |  | 4352 | -0.008 | 0.2672 | No |
| 216 | HERPUD2 |  |  | 4376 | -0.009 | 0.2647 | No |
| 217 | ZNF184 |  |  | 4469 | -0.013 | 0.2543 | No |
| 218 | SATB1 |  |  | 4505 | -0.014 | 0.2506 | No |
| 219 | TAF3 |  |  | 4518 | -0.015 | 0.2494 | No |
| 220 | PIK3R1 |  |  | 4545 | -0.016 | 0.2467 | No |
| 221 | LAS1L |  |  | 4555 | -0.016 | 0.2460 | No |
| 222 | PHF20L1 |  |  | 4576 | -0.017 | 0.2440 | No |
| 223 | KPNA1 |  |  | 4602 | -0.018 | 0.2415 | No |
| 224 | NFATC3 |  |  | 4619 | -0.019 | 0.2400 | No |
| 225 | NPAT |  |  | 4663 | -0.021 | 0.2354 | No |
| 226 | PURA |  |  | 4668 | -0.021 | 0.2354 | No |
| 227 | SRSF11 |  |  | 4687 | -0.021 | 0.2337 | No |
| 228 | KAT6A |  |  | 4783 | -0.025 | 0.2232 | No |
| 229 | ZBTB10 |  |  | 4886 | -0.029 | 0.2120 | No |
| 230 | SEPT6 |  |  | 4896 | -0.029 | 0.2115 | No |
| 231 | ARRDC3 |  |  | 4938 | -0.031 | 0.2074 | No |
| 232 | CNOT9 |  |  | 5053 | -0.035 | 0.1949 | No |
| 233 | RARA |  |  | 5104 | -0.037 | 0.1898 | No |
| 234 | YME1L1 |  |  | 5251 | -0.042 | 0.1738 | No |
| 235 | TRIM69 |  |  | 5332 | -0.046 | 0.1654 | No |
| 236 | KDM5A |  |  | 5365 | -0.047 | 0.1626 | No |
| 237 | MAPK1 |  |  | 5469 | -0.051 | 0.1517 | No |
| 238 | RBPJ |  |  | 5474 | -0.051 | 0.1522 | No |
| 239 | OGFRL1 |  |  | 5490 | -0.052 | 0.1515 | No |
| 240 | BROX |  |  | 5532 | -0.054 | 0.1478 | No |
| 241 | FAM107B |  |  | 5545 | -0.055 | 0.1474 | No |
| 242 | TTC39C |  |  | 5618 | -0.059 | 0.1402 | No |
| 243 | CYB5B |  |  | 5644 | -0.060 | 0.1384 | No |
| 244 | POLR2A |  |  | 5681 | -0.061 | 0.1355 | No |
| 245 | ARHGAP5 |  |  | 5716 | -0.063 | 0.1327 | No |
| 246 | TANK |  |  | 5756 | -0.064 | 0.1294 | No |
| 247 | GABARAPL1 |  |  | 5860 | -0.068 | 0.1188 | No |
| 248 | PPP4R1 |  |  | 5887 | -0.069 | 0.1171 | No |
| 249 | MAX |  |  | 5960 | -0.072 | 0.1102 | No |
| 250 | LYSMD2 |  |  | 6037 | -0.076 | 0.1028 | No |
| 251 | TBCC |  |  | 6091 | -0.079 | 0.0982 | No |
| 252 | ZNF639 |  |  | 6107 | -0.080 | 0.0980 | No |
| 253 | MINDY2 |  |  | 6171 | -0.083 | 0.0923 | No |
| 254 | ADNP |  |  | 6175 | -0.083 | 0.0935 | No |
| 255 | TSNAX |  |  | 6182 | -0.084 | 0.0944 | No |
| 256 | SEPT1 |  |  | 6385 | -0.094 | 0.0729 | No |
| 257 | NETO2 |  |  | 6583 | -0.107 | 0.0522 | No |
| 258 | USP10 |  |  | 6623 | -0.109 | 0.0497 | No |
| 259 | STRN3 |  |  | 6635 | -0.110 | 0.0505 | No |
| 260 | SMIM27 |  |  | 6720 | -0.115 | 0.0430 | No |
| 261 | MIER1 |  |  | 6728 | -0.116 | 0.0443 | No |
| 262 | FYN |  |  | 6823 | -0.123 | 0.0358 | No |
| 263 | HSPH1 |  |  | 6836 | -0.124 | 0.0367 | No |
| 264 | IRF2BPL |  |  | 6859 | -0.126 | 0.0366 | No |
| 265 | ARL4A |  |  | 6930 | -0.131 | 0.0309 | No |
| 266 | UBC |  |  | 6969 | -0.135 | 0.0291 | No |
| 267 | CBX6 |  |  | 7045 | -0.141 | 0.0231 | No |
| 268 | CCDC59 |  |  | 7079 | -0.144 | 0.0220 | No |
| 269 | CITED2 |  |  | 7120 | -0.147 | 0.0201 | No |
| 270 | C6orf62 |  |  | 7221 | -0.158 | 0.0116 | No |
| 271 | IRF2BP2 |  |  | 7287 | -0.166 | 0.0072 | No |
| 272 | RANBP9 |  |  | 7347 | -0.174 | 0.0036 | No |
| 273 | RPA1 |  |  | 7418 | -0.183 | -0.0010 | No |
| 274 | STX10 |  |  | 7429 | -0.185 | 0.0013 | No |
| 275 | YES1 |  |  | 7439 | -0.185 | 0.0037 | No |
| 276 | GNL3 |  |  | 7548 | -0.197 | -0.0050 | No |
| 277 | CDC42EP3 |  |  | 7562 | -0.199 | -0.0028 | No |
| 278 | SNN |  |  | 7577 | -0.201 | -0.0006 | No |
| 279 | BTG3 |  |  | 7602 | -0.204 | 0.0004 | No |
| 280 | EIF4A3 |  |  | 7619 | -0.207 | 0.0024 | No |
| 281 | MXD4 |  |  | 7641 | -0.209 | 0.0039 | No |
| 282 | WTAP |  |  | 7646 | -0.210 | 0.0074 | No |
| 283 | WNK1 |  |  | 7672 | -0.213 | 0.0085 | No |
| 284 | NOSIP |  |  | 7722 | -0.222 | 0.0070 | No |
| 285 | TRIB2 |  |  | 7741 | -0.224 | 0.0091 | No |
| 286 | DCTN6 |  |  | 7760 | -0.226 | 0.0113 | No |
| 287 | C9orf78 |  |  | 7816 | -0.238 | 0.0094 | No |
| 288 | BCAS2 |  |  | 7819 | -0.238 | 0.0136 | No |
| 289 | H2AFX |  |  | 7965 | -0.269 | 0.0020 | No |
| 290 | SNX10 |  |  | 7992 | -0.274 | 0.0041 | No |
| 291 | PKN1 |  |  | 8016 | -0.278 | 0.0066 | No |
| 292 | PSMA1 |  |  | 8109 | -0.299 | 0.0016 | No |
| 293 | ABHD17A |  |  | 8155 | -0.310 | 0.0023 | No |
| 294 | MRPS6 |  |  | 8173 | -0.318 | 0.0063 | No |
| 295 | SRSF3 |  |  | 8191 | -0.323 | 0.0103 | No |
| 296 | SEPT9 |  |  | 8240 | -0.336 | 0.0111 | No |
| 297 | MAZ |  |  | 8394 | -0.387 | 0.0007 | No |
| 298 | SLBP |  |  | 8396 | -0.387 | 0.0078 | No |
| 299 | ARL4C |  |  | 8410 | -0.390 | 0.0136 | No |
| 300 | TPM3 |  |  | 8487 | -0.428 | 0.0129 | No |
| 301 | C12orf75 |  |  | 8546 | -0.456 | 0.0148 | No |
| 302 | HSPE1 |  |  | 8650 | -0.524 | 0.0127 | No |
| 303 | AES |  |  | 8809 | -0.656 | 0.0068 | No |
| 304 | SOD1 |  |  | 8848 | -0.709 | 0.0157 | No |
Table: GSEA details [plain text format]

  

Fig 2: NB\_KI\_CLUSTER8: Random ES distribution      
 Gene set null distribution of ES for **NB\_KI\_CLUSTER8**
